# The Treatment of Fractures

**Published:** 1900-09

**Authors:** 


THE TREATMENT OF FRACTURES.—By Chas. Locke Scudder, M. D., Surgeon to the Mass. General Hospital, Out-Patient Department; Assistant in Clinical and Operative Surgery in the Harvard Medical School. Assisted by Frederic J. Cotton, M. D. With 585 illustrations. Pp. 433. Price, $4.50, net. Philadelphia: W. B. Saunders, 925 Walnut Street. 1900.

From a careful reading of this book one is impressed with its importance as a work of reference in every-day practice. Simplicity is a marked feature throughout the work. The treatment of fractures, as observed in print accompanied with elaborate cuts, is always beautiful to behold and simple enough, but the author discusses the difficulties and makes it clear that he writes from experience. The X-ray has been used extensively in illustrating fractures before and after treatment, a method which, if generally adopted, would teach some better lessons. The author very appropriately discards the words "simple" and "compound," in describing fractures and uses "closed" and "open" instead.

It is a work that may be confidently placed in the library and conscientiously followed in practice. T. J. B.

